# Functional and evolutionary aspects of chemoreceptors

**DOI:** 10.3389/fncel.2012.00048

**Published:** 2012-10-26

**Authors:** Dieter Wicher

**Affiliations:** Department of Evolutionary Neuroethology, Max Planck Institute for Chemical EcologyJena, Germany

**Keywords:** ionotropic receptor, metabotropic receptor, receptor kinase, GPCR, TRP channel

## Abstract

The perception and processing of chemical signals from the environment is essential for any living systems and is most probably the first sense developed in life. This perspective discusses the physical limits of chemoreception and gives an overview on the receptor types developed during evolution to detect chemical signals from the outside world of an organism. It discusses the interaction of chemoreceptors with downstream signaling elements, especially the interaction between electrical and chemical signaling. It is further considered how the primary chemosignal is appropriately amplified. Three examples of chemosensory systems illustrate different strategies of such amplification.

## Introduction

Chemoreceptors transduce an external signal, a volatile molecule (olfaction) or a molecule in solution (gustation) into an intracellular signal. There are two major types of chemoreceptors, ionotropic and metabotropic receptors. Ionotropic receptors (IRs) are ion channels activated by ligand binding. The chemical messenger elicits an immediate electrical signal in the sensory cell. By contrast, activation of metabotropic receptor activates an intracellular signaling cascade which may include enzyme activation, second messenger production or activation of ion channels. Receptor function and sensitivity are usually regulated by interaction with accessory proteins. A high sensitivity of the chemoreceptive machinery results from signal amplification which may take place at various levels of signal processing.

While most chemoreceptors in mammals are metabotropic receptors, chemoreception in insects is ionotropic. The authors of a recent review thus asked whether the choice of a mechanism for a sensory task is “chance or design” (Silbering and Benton, 2010), and concluded that the choice of ionotropic mechanisms for insect chemoreception probably reflects a special mechanistic advantage. For a detailed review on the evolution of insect olfaction see (Hansson and Stensmyr, 2011).

## Physical limits of chemosensation

To detect low pheromone concentrations released in the order of magnitude of ng per hour male insects have expanded the surface area of their antennae (Hansson and Stensmyr, 2011). How are receptor size and resolution related? Analysing bacterial chemotaxis Berg and Purcell (1977) calculated the maximum precision in sensing the concentration of a chemoattractant. The limit is set by the noise due to Brownian motion when sensing a few molecules. *Escherichia coli* can detect amino acids at nanomolar concentration (Mao et al., 2003) which corresponds to only a few molecules per cell volume. The fractional accuracy δ*c*/*c* in determining the concentration *c* is given by:

$$\delta c/c\;=\;1/\sqrt{Drc_{m}t}$$

where *D* is the diffusion coefficient of the chemoattractant, *r* the receptor radius (e.g., cell radius), *c*_m_ the mean concentration and *t* the detection time (Berg and Purcell, 1977). This formula is valid for a single receptor as well as for a receptor array such as a cell surface equipped with receptors (Bialek and Setayeshgar, 2005). To determine a molecule with a diffusion coefficient of 10^−5^ cm^2^/s at a mean concentration of 1 μM with an accuracy of 1%, a cell with a radius of 1 μm requires a measuring time of 10 ms. For a single receptor with a radius of 1 nm a detection time of 17 s would be required. Considering a fixed detection time increasing the receptive surface indeed enhances the resolution as in the above case of pheromone detection.

## Chemosignal processing and amplification

External molecules bind to chemoreceptors located in the plasma membrane, the subsequent receptor activation transduces the external signal across the plasma membrane. For ligand-gated or IRs, binding of the signal molecule opens an ion channel and produces an electrical signal. This process is usually fast (micro to milliseconds). The ion flux changes the membrane potential and thus the electrical activity of the chemosensory neurons. Signals transferred by excitatory IRs are amplified by depolarization which activates various voltage-gated cation channels leading to further depolarizion of OSNs. Another type of amplification may take place when receptor activation leads to Ca^2+^ influx, either directly such as for the Ca^2+^-permeable TRP channels or indirectly when the depolarization activates voltage-gated Ca^2+^-channels. A rise in the free intracellular Ca^2+^ concentration may activate various intracellular signaling cascades thereby amplifying the chemical signal.

For metabotropic receptors binding of the signal molecule initiates changes in intracellular chemical signaling such as enzyme activation and production of second messengers. This process is slower than ionotropic signaling and requires typically 50–150 milliseconds. However, for a tightly packed signaling cascade this delay can be much shorter as in *Drosophila* photoreception where the receptor current activates 20 ms after photon absorption by rhodopsin (Katz and Minke, 2009).

Since metabotropic signaling often involves G protein activation, stimulation of enzymatic activity, and production of second messengers, i.e., processes potentially contributing to a signal amplification, this type of signaling may achieve a high sensitivity of chemodetection. For example, in G protein-coupled receptors (GPCRs) ligand binding activates a number of G proteins setting a first level of signal amplification. This amplification relies on a sufficient long dwelling time of the ligand to the receptor. The residence time of the ligand which is inversely related to the dissociation rate constant is thought to determine the effect of receptor activation under *in vivo* conditions far from thermodynamic equilibrium rather than the affinity defined as the reciprocal of the equilibrium dissociation constant (Tummino and Copeland, 2008). Another mechanism that may restrict the number of activated G proteins is receptor desensitization (Kato and Touhara, 2009). Furthermore, the signal amplification at G protein level is controlled by proteins regulating their activity cycle such as RGS proteins (regulators of G protein signaling), AGS proteins (activators of G protein signaling) and GEFs (guanine nucleotide exchange factors), for example in *C*. *elegans* RGS proteins (Fukuto et al., 2004; Ferkey et al., 2007). G proteins downstream activate enzymes such as adenylyl cyclases. The cAMP production sets the second level of amplification since each molecule may affect further downstream targets such as protein kinases which might add another level of amplification.

Primary electrical signals can elicit secondary chemical signaling, and vice versa, primary chemical signals can be transformed into electrical signals as metabotropic receptors downstream often target ion channels. For example, activation of the vanilloid receptor VR1, a member of the TRP channel family, by capsaicin induces a Ca^2+^ influx in the receptor cell. Ca^2+^ as a universal intracellular signaling molecule can then activate Ca^2+^-dependent proteins such as cyclases and kinases. In mammalian olfactory sensory neurons, odor binding activates the olfactory receptor (OR), a GPCR bound to a stimulatory G_olf_ protein which in turn enhances the cAMP production via adenylyl cyclase stimulation. cAMP binds to and opens cyclic nucleotide gated (CNG) channels which depolarize the neuron and also conduct Ca^2+^. Finally, Ca^2+^ activates Ca^2+^-dependent Cl^−^ channels thereby further depolarizing the cell (Kaupp, 2010). Similarly, receptor guanylyl cyclases catalyze the production of cGMP which also activate CNG channels to depolarize cells and enhance the free Ca^2+^ concentration.

## Special examples for chemosignal amplification

### Bacterial chemoreceptors

In *Escherichia coli*, chemoreceptors transmembrane methyl-accepting chemotaxis proteins (MCPs). They couple via a signal conversion module to histidine autokinases CheA which are inhibited upon chemoattractant and activated upon repellent binding (Hazelbauer et al., 2008). The receptors for attractants bind serine (Tsr), aspartate and maltose (Tar) ribose, glucose and galactose (Trg), and dipeptides (Tap). The phosphorylation state of CheA controls the flagellar motor. The chemoreceptors form dimers which assemble to hexagonally packed trimers. These arrays are concentrated in patches of about 250 nm diameter, corresponding to 1% of the cell surface, and contain nearly half the number of expressed chemoreceptors.

Within the signaling complex chemoreceptor/CheA there is a signal amplification of ~36, i.e., one receptor controls 36 kinase molecules. Moreover, the receptors show strong cooperativity, for attractant stimulation a Hill coefficient of 10 was observed (Hazelbauer et al., 2008). Taken together, the lattice structure of receptor arrangement allows a high degree of interaction within the signaling complexes. This allows the bacteria to detect nanomolar amino acid concentrations (Mao et al., 2003).

### Sea urchin sperm cells

The chemoattractant resact is released from the egg and binds to a transmembrane receptor guanylyl cyclase on a sperm cell. One ligand molecule gives rise to the production of ~45 cGMP molecules (Bönigk et al., 2009). One cGMP molecule is able to activate CNGK, an atypical CNG channel with exclusive K^+^ permeability that forms a pseudotetramer like voltage-gated Na^+^ or Ca^2+^ channels. Conventional CNG channels operate in the micromolar concentration range and are activated in cooperative manner, CNGK operates in the nanomolar range and is activated by binding of a single molecule to the third repeat. CNGK activation produces a transient hyperpolarization followed by activation of voltage-gated Ca^2+^ channels allowing Ca^2+^ to enter the flagellum. At least 25 cGMP molecules were found to be required for eliciting a Ca^2+^ signal. Thus, binding of one chemoattractant molecule is sufficient to produce a number of cGMP molecules that activate the highly cGMP-sensitive CNGK channels which in turn elicit a behavioral response.

### Mammalian olfactory receptors

Odor binding on mouse OR is extremely short. A dwelling time of ~1 ms is not sufficient to activate on average one G protein, similar as previously observed in frog ORs (Bhandawat et al., 2005; Ben-Chaim et al., 2011). Thus, there is no signal amplification at this level. Multiple binding of odor molecules to the same receptor integrates odor signaling, and together with sufficient receptor expression that multiplies binding events and thus the probability of G protein activation leads to a stimulation of adenylyl cyclase III. This raises the cAMP level to activate CNG channels thereby depolarizing the cell and importing Ca^2+^. The signal amplification takes place when Ca^2+^ activates Ca^2+^-dependent Cl^−^ channels which further depolarize the cell (Kaupp, 2010).

The delay between binding of odor molecules to the receptor and the development of the receptor potential depends on the odor concentration and can range between 100 ms at submicromolar concentration and 25–40 ms at concentrations up to 100 μM (Ghatpande and Reisert, 2011). A delay in this order of magnitude seems to be not limiting since a behavioral response appears already 200 ms after odor stimulation (Abraham et al., 2004).

## Chemoreceptor types in evolution

From bacteria to men both types of receptors, metabotropic and ionotropic, are used to perceive chemical signals (Table 1) (Biswas et al., 2009; Gees et al., 2010; Nordström et al., 2011). Most of them are conserved during evolution, yet there are cases of receptor gene degeneration such as the CO_2_-sensing guanylyl cyclase D in primates (Young et al., 2007) or TRPC2, a channel part of the pheromone signaling cascade, in men (Zufall, 2005).

**Table 1:** **Chemoreceptors in model organisms operating via a metabotropic or an ionotropic mechanism**.

	**Metabotropic**			**Ionotropic**	
	**RC/RK**	**GPCR**	**GR/OR**	**TRP**	**IR**
*E. coli*	X	−	−	−	X
*S. cerevisae*	−	X	−	X	−
*C. elegans*	X	X	X	X	X
*D. melanogaster*	−	−	X^*^	X	X
*M. musculus*	X	X	−	X	−

RC/RK, receptor cyclase/receptor kinase; GPCR, G protein-coupled receptor; GR/OR, gustatory receptor, olfactory receptor; TRP, ion channel family named according to the first member to be discovered (“transient receptor potential” channel in Drosophila photoreceptors); IR, “ionotropic receptor”, a variant ionotropic glutamate receptor protein serving as olfactory receptor.

* GR/ORs in insects are 7-TM proteins as GPCRs, but are inversely oriented in the membrane. They do not belong to the GPCR superfamily according to a bioinformatics analysis (Benton et al., 2006; Nordström et al., 2011), and form ionotropic receptors. X, receptor type reported for that species.

Bacteria obtain nutrient information for chemotaxis from receptor-associated kinases (Hazelbauer et al., 2008) and sense amino acids using precursors of ionotropic glutamate receptors (iGluRs, Chiu et al., 1999). Yeast express GPCRs to detect sugar and pheromones (Versele et al., 2001) as well as TRP channels for sensing aromatic compounds (Nilius and Owsianik, 2011). Highly sugar-sensitive receptors are the 12-TM proteins Snf3/Rgt2 which regulate the expression of sugar transporters (Gancedo, 2008).

In more complex organisms, specialized chemosensory neurons detect volatile or external chemical messengers in solution. Receptor activation induces a change in the electrical activity of sensory neurons. IRs form ion channels gated by ligand binding to the receptor. Metabotropic receptors couple to intracellular signaling systems regulating the activity of targets such as ion channels thereby changing the neuronal activity.

In nematodes metabotropic chemoreceptors comprise receptor guanylyl cyclases and a large number of GPCRs (Bargmann, 2006). Receptors operating via an ionotropic mechanism are TRP channels (Nilius and Owsianik, 2011), and “Ionotropic Receptors” (IRs), ORs sensing general odors which are related to iGluRs (Croset et al., 2010).

In insects, ORs are heterodimers composed of two proteins with 7-transmembrane topology like GPCRs (Neuhaus et al., 2005). However, there is no sequence similarity to other GPCRs and the proteins are inversely oriented in the membrane (Benton et al., 2006). One of these proteins is odor-specific, the other one a co-receptor with chaperone function (Larsson et al., 2004). While there are indications that odors initiate metabotropic signaling (Wicher et al., 2008; Deng et al., 2011), the primary odor response is ionotropic (Sato et al., 2008; Wicher et al., 2008). Other IRs in insects are gustatory receptors [GRs, (Sato et al., 2011), IRs (Benton et al., 2009), and TRP channels (Nilius and Owsianik, 2011)].

In mammals metabotropic chemoreceptors comprise receptor tyrosine kinases (Petersen et al., 2011), guanylyl cyclases (Fülle et al., 1995; Sun et al., 2009), and a large number of GPCRs (Kaupp, 2010). Most olfactory and pheromone receptors are GPCRs (Fleischer et al., 2009). Taste receptors for sweet, bitter, and umami are GPCRs whereas those for sour and salty are thought to be ionotropic (Chandrashekar et al., 2006). Receptors for hot and spicy compounds as capsaicin, for cool compounds as menthol or pungent compounds as mustard oil are also ionotropic TRP channels (Damann et al., 2008). IRs are not expressed in mammals (Croset et al., 2010). Instead, mammalian central synapses express various subtypes of the related iGluRs.

## Conclusion

Chemosensory systems are functional complexes of receptors and downstream signaling elements. There seems to be no preference for the use of either metabotropic or IRs for the detection of chemicals during evolution. Moreover, receptors for a given sense might be both metabotropic and ionotropic as seen above for mammalian taste receptors. There is a pronounced conservation of chemoreceptors during evolution. It is rather rare that receptors disappear at a certain stage like the IRs in vertebrates. As seen in mammalian ORs, there is no selection toward perfection. It is possible to neglect the potential of G proteins for a signal amplification. Although the chemosensory system was not optimized for fast processing, the organism as a whole is capable of behaviorally responding to odor stimulation in astonishingly short time. A high sensitivity of chemosensory systems can be achieved by spatial arrangements such as the chemosensory patches in bacteria which provide the basis for high cooperativity of signaling elements. On the other hand, the capability of sea urchin sperm cells to react to single resact molecules demonstrates that high sensitivity can also be obtained in a remarkably simple way unless the internal signaling cascade allows a sufficiently high amplification of the external signal.

### Conflict of interest statement

The author declares that the research was conducted in the absence of any commercial or financial relationships that could be construed as a potential conflict of interest.
